# Association of maternal postpartum depression symptoms with infant neurodevelopment and gut microbiota

**DOI:** 10.3389/fpsyt.2024.1385229

**Published:** 2024-05-21

**Authors:** Lepeng Zhou, Linghong Tang, Chuhui Zhou, Shi Wu Wen, Daniel Krewski, Ri-hua Xie

**Affiliations:** ^1^ School of Nursing, Southern Medical University, Guangzhou, Guangdong, China; ^2^ Women and Children Medical Research Center, Foshan Women and Children Hospital, Foshan, Guangdong, China; ^3^ Clinical Epidemiology Program, Ottawa Hospital Research Institute, Ottawa, ON, Canada; ^4^ School of Epidemiology and Public Health, University of Ottawa, Ottawa, ON, Canada; ^5^ Department of Obstetrics and Gynecology, University of Ottawa, Ottawa, ON, Canada; ^6^ Risk Science International, Ottawa, ON, Canada

**Keywords:** postpartum depression, neurodevelopment, gut microbiome, gut metabolome, gut-brain axis

## Abstract

**Introduction:**

Understanding the mechanisms underlying maternal postpartum depression (PPD) and its effects on offspring development is crucial. However, research on the association between maternal PPD, gut microbiota, and offspring neurodevelopment remains limited. This study aimed to examine the association of maternal PPD symptoms with early gut microbiome, gut metabolome, and neurodevelopment in infants at 6 months.

**Methods:**

Maternal PPD symptoms were assessed using the Edinburgh Postpartum Depression Scale (EPDS) at 42 days postpartum. Infants stool samples collected at 42 days after birth were analyzed using 16S rRNA sequencing and liquid chromatography–mass spectrometry (LC–MS) detection. Infant neurodevelopment was measured at 6 months using the Ages and Stages Questionnaire, Third Edition (ASQ-3). Correlations between gut microbiota, metabolites and neurodevelopment were identified through co-occurrence network analysis. Finally, mediation analyses were conducted to determine potential causal pathways.

**Results:**

A total of 101 mother-infant dyads were included in the final analysis. Infants born to mothers with PPD symptoms at 42 days postpartum had lower neurodevelopmental scores at 6 months. These infants also had increased alpha diversity of gut microbiota and were abundant in *Veillonella* and *Finegoldia*, while depleted abundance of *Bifidobacterium*, *Dialister*, *Cronobacter* and *Megasphaera.* Furthermore, alterations were observed in metabolite levels linked to the Alanine, aspartate, and glutamate metabolic pathway, primarily characterized by decreases in N-Acetyl-L-aspartic acid, L-Aspartic acid, and L-Asparagine. Co-occurrence network and mediation analyses revealed that N-Acetyl-L-aspartic acid and L-Aspartic acid levels mediated the relationship between maternal PPD symptoms and the development of infant problem-solving skills.

**Conclusions:**

Maternal PPD symptoms are associated with alterations in the gut microbiota and neurodevelopment in infants. This study provides new insights into potential early intervention for infants whose mother experienced PPD. Further research is warranted to elucidate the biological mechanisms underlying these associations.

## Introduction

Postpartum depression (PPD) is a prevalent mental health issue in the perinatal period, affecting approximately 17.2% of mothers globally, with rates varying from 6.5% to 60.9% across different populations (1). This condition may emerge during pregnancy or persist beyond the first postpartum month, possibly lasting several years. PPD not only jeopardizes the physical and mental well-being of mothers, but is also linked to adverse impacts on the development and behavior of infants (2). Previous studies have shown that postpartum depressive symptoms were associated with delayed early child development (3) and reduced verbal Intelligence Quotient (IQ) in offspring (4). Similar findings have been observed in animal models, where offspring from stressed dams had increased anxiety-like behavior and behavioral deficits compared to those from non-stressed mothers (5, 6). Elucidating the potential biological mechanisms behind these associations is the key to the development of strategies for early intervention in at-risk infants.

The early stages of life represent a critical window for gut microbial colonization, influencing infant health in terms of growth, neurodevelopment, and temperament (7, 8). Factors such as maternal emotional status can shape the composition of the infant’s early-life gut microbiota. Research indicates that infants born to mothers with higher reported levels of anxiety and perceived stress had less alpha diversity and lower abundance of *Bifidobacterium* dentium (9). Furthermore, maternal depressive symptoms have been linked to reduced fecal Immunoglobulin A concentrations in infants (10). However, longitudinal studies examining the infant gut microbiota in mediating the effects of maternal PPD on infant health outcomes are lacking.

Therefore, we hypothesize that infants born to mothers with postpartum depressive symptoms are subject to early gut microbiota composition and influencing neurodevelopment. Leveraging data from our previous randomized controlled trial (11), this study aims to investigate the association of maternal PPD symptoms with infant gut microbiota and neurodevelopment.

## Materials and methods

### Study participants

This study was derived from a triple-blinded randomized controlled trial investigating the effects of vaginal microbiota transfer on the gut microbiome and early neurodevelopment of infants born by cesarean delivery (11). Vaginal delivery cases during the same period served as natural controls. Pregnant women were enrolled from December 2020 to July 2021 at a tertiary teaching hospital in Guangdong, China. Eligible participants were women aged 18 years or older, with singleton intrauterine pregnancies, and newborns who were alive with a gestational age of over 37 weeks. Women were excluded if they were infected with sexually transmitted diseases (STDs), including syphilis, HIV, gonorrhea, and chlamydia. This study enrolled mothers regardless of their delivery mode. Newborns with Apgar scores less than seven at one minute after birth, as well as those with any congenital anomalies or intrauterine infections, were excluded. Given that the focus of this study was to explore the effects of maternal PPD symptoms on infant gut microbiome and neurodevelopment, pregnant women with a history of mental disorders before or during pregnancy, or those using psychiatric medications in the preceding 6 months were also excluded. Informed consent was obtained from all participants. A total of 101 participants were enrolled in the study and completed baseline assessments, irrespective of their group allocations in the randomized trial.

### Outcome measures

We assessed infant development using the Ages and Stages Questionnaire, Third Edition (ASQ-3) (12) at 6 months after birth. The assessment covers five domains: communication, problem-solving, gross motor, fine motor, and personal social skills, for a total of 30 age-appropriate items. Each item is assigned a score of 10 if the child has mastered the skill, 5 if the skill is emerging or occasional, and 0 if the child cannot master the skill. The individual domain scores are obtained by summing scores of 6 items, resulting in a range of 0 to 60 for each domain. The total ASQ-3 score is the sum of the five domain scores, yielding a total score range from 0 to 300.

### Exposure measures

The primary exposure was maternal PPD symptoms at 42 days postpartum, assessed using the Edinburgh Postpartum Depression Scale (EPDS) (13), thereby establishing two distinct groups: the PPD group and the non-PPD group. Depressive symptoms were measured using the 10-item EPDS, which describes depression as cognitive and affective features that last for at least one week (13). Each item has four possible responses and is scored from 0 to 3; individual item scores are then summed to generate a total score ranging from 0 to 30. Consistent with prior research validating a cutoff score of 11 for better sensitivity to major or minor depression (13–15), scores of 11 or higher were considered indicative of depressive symptoms.

### Potential confounders

Potential confounders evaluated included maternal and infant characteristics that have an established or potential associations with infants’ neurodevelopment and/or maternal PPD symptoms. According to previous reports, we considered several maternal and infant characteristics as covariates, including maternal pre-pregnancy BMI (16), weeks of gestation (1, 17), birth mode (vaginal delivery, cesarean section, cesarean section exposed to maternal vaginal fluids) (18), neonatal intensive care unit (NICU) care (19), infant feeding (exclusive breastfeeding, formula feeding, and partial breastfeeding at 42 days postpartum, along with the introduction of complementary foods at 6 months) (20). Demographic and clinical information about the study participants was collected from the transcribed medical records and obtained through a self-administered questionnaire.

### Sample collection and gut microbiota sequencing

Infant fecal samples were collected at 42 days after birth. A sterile applicator stick was used to collect stool from a soiled diaper, placing it directly into a sterile collection tube. Samples were then refrigerated until transferred to a -80 °C freezer, where they were stored until sequencing procedures were performed. Genomic DNA was extracted from infant stool using MoBio PowerSoil DNA Isolation Kits (MoBio Laboratories, Carlsbad, CA, USA) in accordance with the manufacturer’s instructions. Amplicon sequencing of the V3-V4 gene region of the 16S ribosomal RNA was conducted, following the procedures detailed elsewhere (11).

### Metabolomics profiling

Targeted liquid chromatography–mass spectrometry (LC–MS) was used to characterize the fecal metabolomic signatures as our previously published study (11). Briefly, dried fecal samples were homogenized, extracted, and derivatized. Subsequently, supernatant aliquots underwent ultra-performance liquid chromatography coupled to tandem mass spectrometry. The LC–MS system, controlled by MassLynx 4.1 software, employed an ACQUITY BEH C18 column. A mixed standard solution calibrated the absolute concentrations of metabolites, processed using Targeted Metabolome Batch Quantification (TMBQ) software.

### Statistical analyses

Participant characteristics in the PPD and non-PPD groups were compared at 42 days postpartum using appropriate statistical tests. Linear regression analyses were conducted to explore the associations between maternal PPD symptoms and neurodevelopmental outcomes in infants. In the univariate linear regression model, maternal PPD symptoms was the independent variable and ASQ-3 total score and the scores for its 5 domains were the dependent variables. The multivariable linear regression model considered confounders including pre-pregnancy BMI, gestational weeks, delivery mode, NICU care, and complementary food at 6 months. The delivery mode covariate adjusted in this study included categories from the original trial (vaginal delivery, cesarean section, cesarean section with exposure to vaginal fluids) (11). To reduce potential bias from missing data, particularly regarding complementary food intake at 6 months and ASQ-3 scores, we employed a random forest imputation method to estimate and impute the missing values in the dataset. A sensitivity analysis was conducted to assess the robustness of the association between maternal PPD symptoms and infant neurodevelopment after imputation.

Microbiome alpha diversity evenness was assessed using the Shannon diversity index and compared between the PPD and non-PPD groups through the Wilcoxon rank-sum test. Differences in community-level composition, as measured by unweighted UniFrac distances matrices between the two groups, were assessed using permutation multivariate analysis of variance (PERMANOVA) with the adonis2 function (vegan package) (21).

The Linear Discriminant Analysis Effect Size (LEfSe) algorithm was used to identify differentially abundant taxa between groups with a logarithmic linear discriminant analysis (LDA) score cutoff of 2. Multivariate linear regression was used to identify statistically significant associations between microbial features and maternal PPD symptoms. The analyses were adjusted for mode of delivery (including original intervention for CS) and feeding type at 42 days. The discriminative fecal metabolites between the two groups were identified using the Wilcoxon rank-sum test. Pathway enrichment analysis was carried out based on the Kyoto Encyclopedia of Genes and Genomes (KEGG) Pathway Database (http://www.genome.jp/kegg/) and MetaboAnalyst database (http://www.metaboanalyst.ca/).

A co-occurrence network, comprising discriminative fecal genera, metabolites, and neurodevelopmental domain scores, was constructed using Spearman’s correlation analysis and visualized in Cytoscape (Version 3.2.1, http://www.cytoscape.org). Causal mediation analysis was used to test whether maternal PPD symptoms at 42 days postpartum influenced infant neurodevelopment at 6 months through intermediate gut microbiota or metabolites (22) with the above mentioned confounders adjusted. *P*-values were adjusted based on the Benjamini–Hochberg false discovery rate (FDR) method, with the significance level and FDR threshold set at 0.05 and 0.25, respectively. All analyses were performed using R version 4.2.1.

## Results

### Maternal and infant characteristics

**Figure 1:**
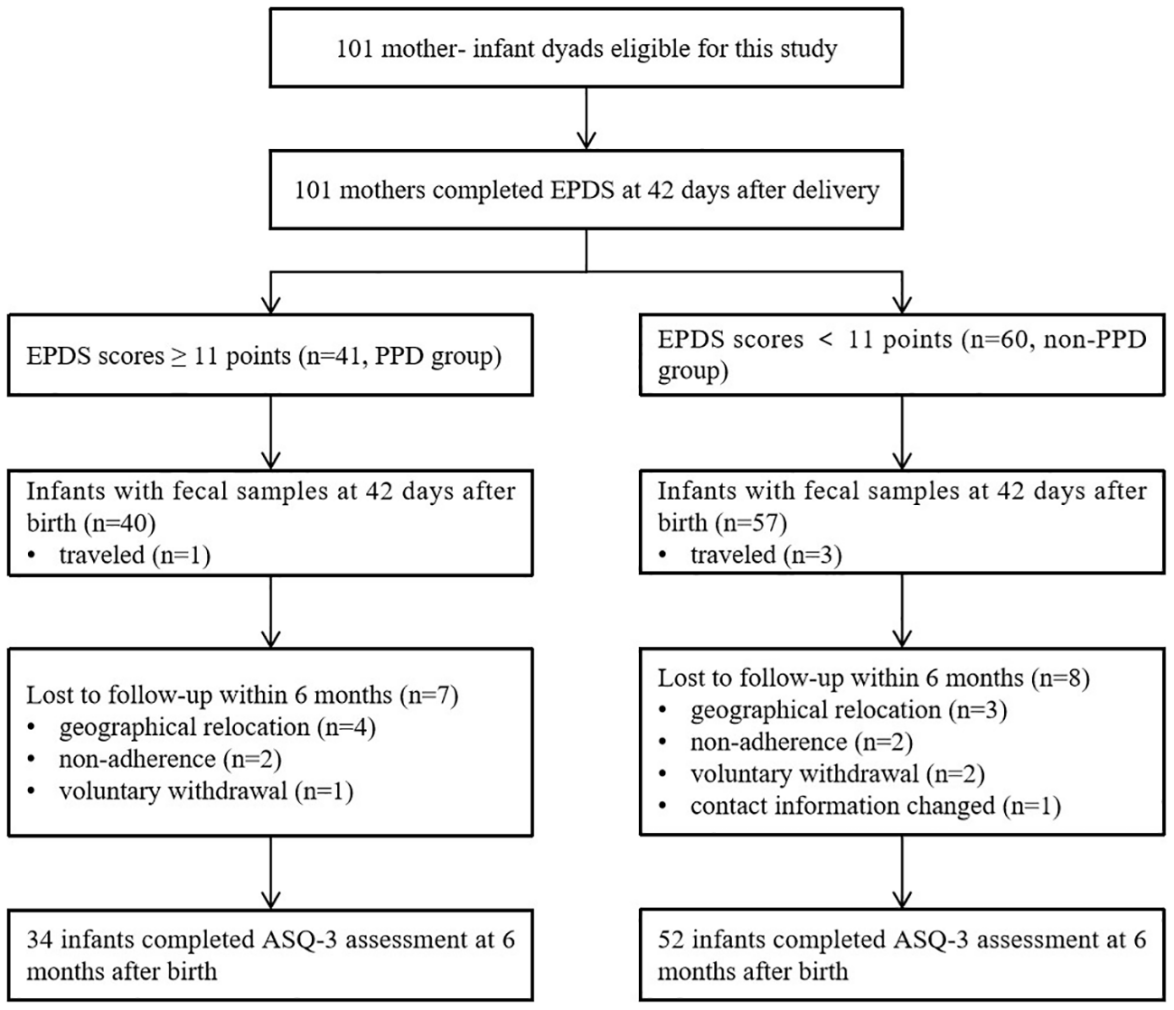
Flowchart of mother-infant dyads included in the study.

A total of 101 mothers who met the inclusion criteria were enrolled in the study, and all of them completed the depression assessment online through a web-based platform using the EPDS at 42 days postpartum. Among them, 4 mother-infant dyads were temporarily residing outside our local area, leaving 97 infant fecal samples being were collected at 42 days after birth. However, within the first 6 months, a total of 15 infants were lost to follow-up, leaving 86 infants available for neurodevelopmental evaluation using ASQ-3 (**Figure 1**).

A total of 41 mothers experienced PPD at 42 days after delivery, based on a cutoff score of 11 on the EPDS. Of these, 40 mothers contributed infant fecal samples for subsequent gut microbiota analysis. The demographic and clinical characteristics of the mothers and infants are presented in **Table 1**. The participants were divided into two groups based on their EPDS scores at 42 days after delivery (PPD group vs. non-PPD group), representing mothers with and without postpartum depression. The age range of mothers was 19 to 42 years old, with the majority being multiparous women. No infants were received antibiotic within the first 42 days of life due to physical illness. No significant differences were observed in occupation, education, income, pre-pregnancy BMI, gestational age, mode of delivery, and feeding types between the two groups. A comparable number of infants in both the non-PPD (24 infants) and PPD (8 infants) groups had received maternal vaginal microbiota transplantation at birth as part of our previous RCT (11). At the 6-month follow-up, 34 infants in the PPD group and 52 in the non-PPD group completed the neurodevelopment assessment using ASQ-3.

**Table 1:** Maternal and infant demographic and clinical characteristics between the non-PPD and PPD groups.

Maternal and infant characteristics	non-PPD^a^ (n=60)	PPD^a^ (n=41)	*P* value
Maternal characteristics			
Maternal age (years), mean ± SD	30.4 (4.9)	29.1 (6.0)	0.229
Marital status			0.077
Married	59 (98.3)	36 (87.8)	
Unmarried	1 (1.7)	5 (12.2)	
Occupation			0.284
Professional	11 (18.3)	4 (9.8)	
Company or factory worker	14 (23.3)	14 (34.2)	
Self-employed	18 (30.0)	8 (19.5)	
Housewife	17 (28.3)	15 (36.6)	
Education			0.072
Junior high school or below	19 (31.7)	22 (53.7)	
High school	20 (33.3)	11 (26.8)	
College or above	21 (35.0)	8 (19.5)	
Monthly household income per capita, RMB			0.740
<5000	20 (33.3)	15 (36.6)	
5000–8000	22 (36.7)	12 (29.3)	
>8000	18 (30.00)	14 (34.2)	
Primipara			0.061
No	48 (80.0)	25 (61.0)	
Yes	12 (20.0)	16 (39.0)	
Pre-pregnancy BMI, kg/m^2^	21.5 (2.6)	21.1 (3.2)	0.477
Gestational weight gain, kg	13.8 (4.2)	14.3 (4.5)	0.602
Excessive gestational weight gain			0.708
No	40 (66.7)	25 (61.0)	
Yes	20 (33.3)	16 (39.0)	
Perinatal antibiotic use			0.334
No	14 (23.3)	14 (34.1)	
Yes	46 (76.7)	27 (65.9)	
Infant characteristics			
Gestational age (days), mean ± SD	272.4 (7.5)	274.1 (6.6)	0.241
Birth weight (kg), mean ± SD	3.2 (0.4)	3.3 (0.4)	0.290
Mode of delivery			0.088
VD	18 (30.0)	15 (36.6)	
CS	18 (30.0)	18 (43.9)	
CS+VMT^b^	24 (40.0)	8 (19.5)	
Gender			>0.999
Boy	37 (61.7)	25 (61.0)	
Girl	23 (38.3)	16 (39.0)	
NICU care within the first 28 days			0.148
Yes	8 (13.3)	11 (26.8)	
No	52 (86.7)	30 (73.2)	
Hospitalization^c^			0.650
Yes	2 (3.3)	0 (0.0)	
No	58 (96.7)	41 (100.0)	
Feeding type at 42 days			0.410
Exclusive breastfeeding	40 (66.7)	25 (61.0)	
Formula feeding	5 (8.3)	7 (17.1)	
Partial breastfeeding	15 (25.0)	9 (22.0)	
Complementary food at 6 months			0.260
No	11 (18.3)	3 (7.3)	
Yes	38 (63.3)	31 (75.6)	
Missing data	11 (18.3)	7 (17.1)	

a Data are reported as n (%) unless otherwise indicated.

b These infants born via CS were exposed to maternal vaginal microbiota in our original RCT (11).

c The data were collected from 28 days to 42 days after birth.

SD, standard deviation; BMI, body mass index; VD, vaginal delivery; CS, cesarean section; VMT, vaginal microbiota transfer; NICU, neonatal intensive care unit.

### Association between maternal PPD symptoms and infant neurodevelopment at 6 months

**Figure 2:**
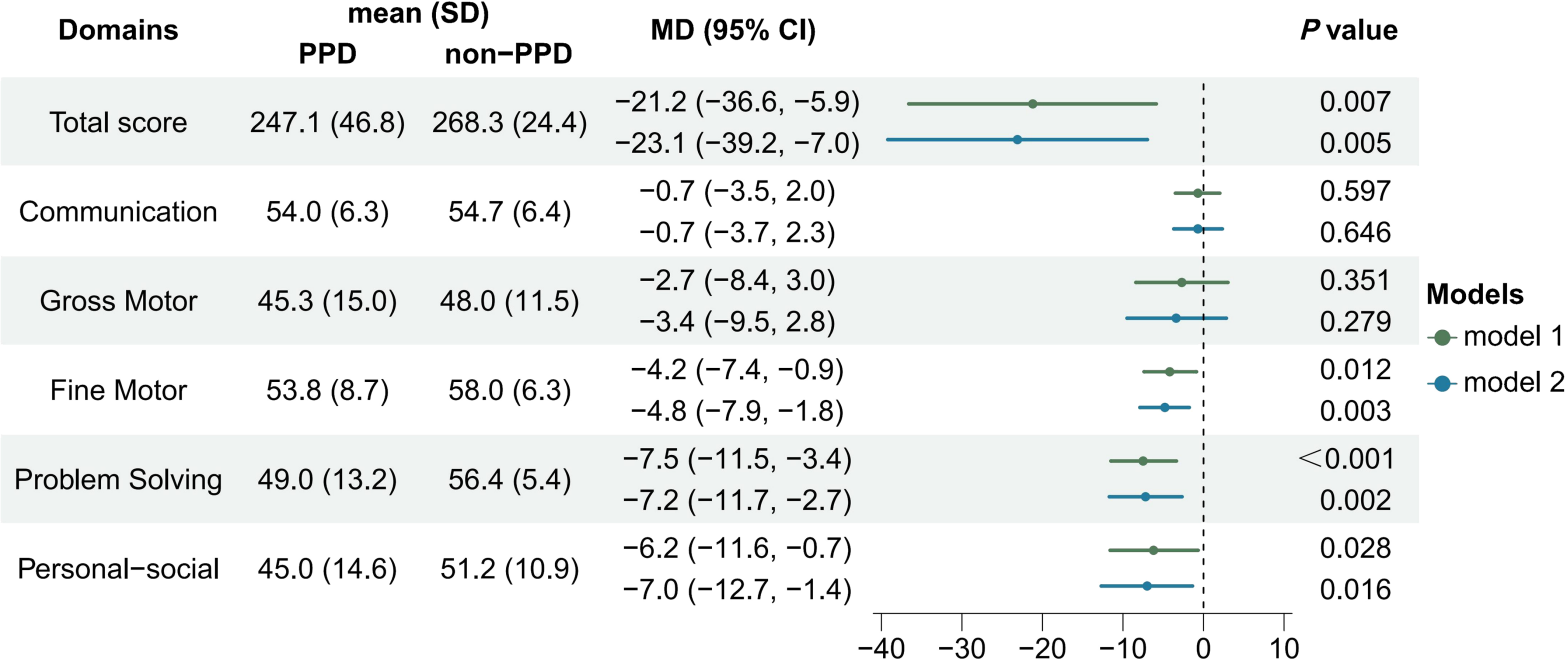
Comparison of infant neurodevelopment between PPD and non-PPD groups based on ASQ-3 scores at 6 months after birth. [Model 1 was the crude model, model 2 was adjusted for pre-pregnancy BMI, gestational age, NICU care, mode of delivery (intervention factor included), and introduction of complementary food at 6 months.].

The linear regression results showed that infants in the PPD group had significantly lower scores in ASQ-3 total score (MD = -21.2; 95% CI = -36.6 to -5.9; *P*=0.007), fine motor (MD = -4.2; 95% CI = -7.4 to -0.9; *P*=0.012), problem-solving (MD = -7.5; 95% CI = -11.5 to -3.4; *P*<0.001) and personal-social domains (MD = -6.2; 95% CI = -11.6 to -0.7; *P*=0.028). These differences remained statistically significant in multiple linear regression models after adjusting for confounders, including maternal pre-pregnancy BMI, gestational age, mode of delivery (including original CS intervention factor), NICU care of infants within 42 days of birth, and introduction of complementary food at 6 months after birth (**Figure 2**). After imputing the missing data with random forest methods, the significant results of the multiple regression model remained.

### Association between maternal PPD symptoms and infant gut microbiota

**Figure 3:**
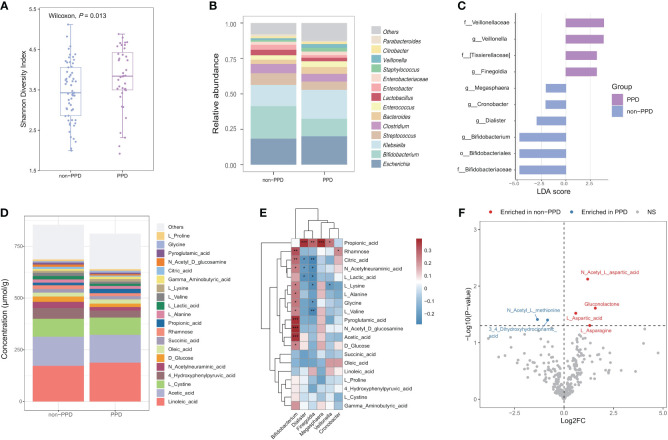
Associations between maternal PPD symptoms at 42 days and infant gut microbiota and metabolome at 42 days after birth. **(A)** Comparison of Shannon diversity index between infants in the PPD and non-PPD groups at 42 days using Wilcoxon test. **(B)** Relative abundances of the 14 predominant genera between groups. Dissimilarities in gut microbiota composition at genus level between PPD and non-PPD groups at 42 days. **(C)** LEfSe (LDA>2) illustrating taxa that are significantly different in infants between PPD and non-PPD groups. **(D)** Metabolite composition of infants in non-PPD and PPD groups. The metabolites with the top 20 concentrations are shown. **(E)** Heatmap of associations between differentially abundant genera and the top 20 concentrations metabolites (Spearman’s rank correlation analysis). *** *P*<0.001, **P<0.01, *P<0.05 (FDR<0.25). **(F)** Volcano chart of fold changes in metabolites in stool samples of infants in the PPD and non-PPD groups. The dotted line represents *P* = 0.05. Red dots show enriched metabolites in non-PPD group, while blue dots show opposite. Notable metabolites are labeled.

We examined the associations between infant gut microbiota and maternal PPD symptoms using infant stool samples collected at 42 days after birth. Our findings revealed that infants exposed to maternal PPD symptoms had higher Shannon diversity than those in the non-PPD group at 42 days (*P*=0.013, **Figure 3A**); the maternal EPDS score at day 42 was also positively associated with the Shannon diversity index (r=0.22, *P*=0.032). However, the overall microbial community composition did not significantly differ between the PPD and non-PPD groups (PERMANOVA test, *P*=0.332). The predominant phyla across all samples were Proteobacteria, Firmicutes, Actinobacteria, and Bacteroidetes. At the genera level, 14 dominant genera, constituting over 90% of the total microbial abundance, were listed (**Figure 3B**). Among these, *Escherichia*, *Bifidobacterium*, and *Klebsiella* were the three most abundant genera in infants. A total of 10 taxa showed significant changes in abundance with the criteria LDA>2.0 and *P*<0.05 (**Figure 3C**). Notably, the abundance of genera *Bifidobacterium*, *Dialister*, *Cronobacter* and *Megasphaera* was higher in the non-PPD group, while *Veillonella* and *Finegoldia* were enriched in the PPD group. When performing multiple linear regression after adjusting for mode of delivery (including original CS intervention factor) and feeding type at day 42 to identify differential abundant taxa between groups, we observed that *Dialister* (*P*=0.047) and *Blautia* (*P*=0.038) were significantly enriched in infants exposed to maternal PPD symptoms, while *Bifidobacterium* (*P*=0.048) was significantly depleted.

### Association between maternal PPD symptoms and infant gut metabolites

Next, we performed targeted metabolomic profiling of fecal samples from infants in the non-PPD and PPD groups by using liquid chromatograph-mass spectrometry (LC–MS). Infants in the non-PPD group harbored a number of richer and higher-concentration metabolites in stool (**Figure 3D**). The top 3 metabolites in infants at 42 days were Linoleic acid, Acetic acid and L-Cystine. Next, we focused on the correlations between differential microbial features and the top 20 metabolites (**Figure 3E**). Impressively, positive correlations were identified between the abundance of *Bifidobacterium* and the majority of fecal metabolites, whereas the abundance of *Finegoldia* exhibited negative correlations. By using the Wilcoxon test, six discriminative metabolites between PPD and non-PPD groups were identified (**Figure 3F**). Specifically, compared to those in the non-PPD group, the PPD group displayed enrichment of 2 metabolites, 3,4-Dihydroxyhydrocinnamic acid and N-Acetyl-L-methionine, and depletion of 4 metabolites, including N-Acetyl-L-aspartic acid, Gluconolactone, L-Aspartic acid, L-Asparagine. Moreover, pathway-based differential abundance analysis highlighted that the metabolic pathway of Alanine, aspartate and glutamate metabolism was significantly downregulated in the PPD group.

### Association of infant gut microbiota and gut metabolites with neurodevelopment at 6 months of age

**Figure 4:**
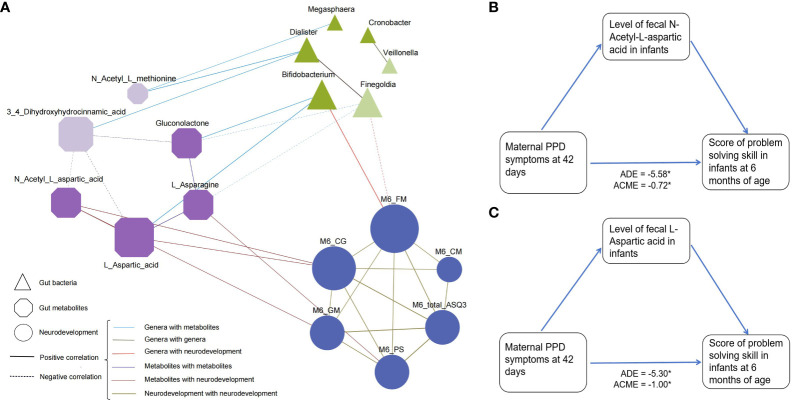
Association between differentially abundant genera, metabolites, and infant neurodevelopment at 6 months of age. **(A)** A co-occurrence network constructed from the relative abundances of differential microbial genus, fecal metabolites as well as neurodevelopment score in infants in PPD group and non-PPD group. The spearman correlation analysis was used to explore the co-occurrence network. The node size signifies its degree, representing the number of network connections. The shape of node denotes the components (ellipse: neurodevelopment domains, triangle: microbial genera, and cut-rectangle: metabolites). Deep and light colors indicate increased and decreased relative abundances in non-PPD compared to PPD, respectively. Solid and dashed edges represent positive and negative correlations, respectively. The colors and shapes of nodes indicate the categories of nodes. Edges thickness indicate the magnitude of correlation. FM, fine motor; GM, gross motor; CM, communication; CG, problem solving skills; PS, personal-social skills. **(B, C)** Mediation analysis assessing the role of **(B)** N-Acetyl-L-aspartic acid and **(C)** L-Aspartic acid in the association between maternal PPD symptoms and infant problem-solving skill scores. The model was adjusted for gestational age, mode of delivery, feeding type at day 42, NICU care, and maternal pre-pregnancy BMI.

To explore the potential relevance between bacterial composition and metabolomic phenotypes, we calculated Spearman’s correlation matrices and constructed a co-occurrence network of differential microbial genera, metabolites and neurodevelopment (**Figure 4A**). Co-occurrence network analysis revealed a total of 36 co-occurrence relationships, with 16 connections found in microbiota-metabolite. Of these, 5 were negative, with *Finegoldia* and 3,4-Dihydroxyhydrocinnamic acid being the major contributors to this finding, which were also enriched in the PPD group. Interestingly, *Bifidobacterium* had positive relationships with Gluconolactone and L-Asparagine, while *Finegoldia* showed opposite. When linking these differential genera and metabolites with infant neurodevelopment, the *Bifidobacterium* was the only taxa showing positive correlation with infant fine motor skill, while *Finegoldia* showed opposite. Moreover, the metabolites involved with Alanine, aspartate and glutamate metabolism were positively correlated with neurodevelopment. For example, L-Aspartic acid and L-Asparagine were positively associated with personal-social score, L-Aspartic acid and N-Acetyl-L-aspartic acid were positively associated with problem solving score, L-Aspartic acid was positively associated with fine motor.

Furthermore, to evaluate whether metabolites can mediate the microbial impact on the association between maternal PPD symptoms at day 42 postpartum and infant neurodevelopment, we performed mediation analyses using the above identified discriminative metabolites. Results indicated that the level of fecal N-Acetyl-L-aspartic acid and L-Aspartic acid mediated the relationships between maternal PPD symptoms at 42 days postpartum and infant problem-solving skill scores, with an effect size of -0.72 (95% CI = -1.59 to -0.11, **Figure 4B**) and -0.34 (95% CI = -2.42 to -0.08, **Figure 4C**), respectively.

## Discussion

The prevalence of PPD in this study population was approximately 40%, which is higher than in other studies (1, 23, 24). Previous research has indicated that cesarean section delivery could elevate the risk of PPD (25, 26). In this study, there were twice as many women who delivered by cesarean section compared to those who delivered vaginally. Furthermore, our definition of PPD differs from those of other studies due to variations in the use of the EPDS tool and its cut-off points. Even so, we cannot ignore the global trend of increasing PPD incidence and its harm. With escalating rates of maternal perinatal depression, we face not only the risk of maternal mental disorders but also growing concerns about the potential long-term impacts on infant health and well-being. A particular emphasis in research on infant neurodevelopment has been on the early development of gut microbiota (27–29). It is therefore crucial to explore the associations among maternal PPD, infant gut microbiota, gut metabolites, and neurodevelopment. In this study, we found that maternal PPD symptoms at day 42 postpartum were negatively associated with infant neurodevelopment at 6 months of age. This association could be partially explained by alterations in the infant’s gut microbiota and gut metabolites.

Maternal psychiatric disorders and stress-induced disturbances in bacterial community structure have been confirmed in both human and preclinical animal studies (5, 30–32). Our data further indicated that maternal PPD symptoms were correlated with changes in the microbial composition of offspring within the first 6 weeks after birth. Infants born to mothers with depressive symptoms had higher alpha diversity in their gut microbiota and were characterized by a lower relative abundance of *Bifidobacterium*. These findings were consistent with a recent study reporting that both maternal prenatal and PPD could affect offspring gut microbiome diversity and *Bifidobacterial* abundances (9). However, the measurement of maternal PPD and the collection of infant fecal samples were conducted simultaneously in our study. Despite this synchrony, it is essential to recognize that the EPDS evaluates depressive symptoms over the past week, whereas PPD typically manifests within the first 30 days postpartum (2, 33). Consequently, it is conceivable that maternal alterations in mood and behavior may have already exerted an influence on the developing infant gut microbiota prior to the measurement at 42 days. Future studies with longitudinal designs are warranted to elucidate the causal relationships between maternal PPD and infant gut microbiota dynamics.

The mechanism by which early-life exposure to maternal PPD influences infant gut microbiota and neurodevelopment is still unclear. One significant factor is that exposure to maternal depression can affect the bacterial composition of breast milk at both the phyla and genera levels (34), an important observation considering that breast milk is a primary dietary and microbial source for the offspring. Moreover, PPD may affect infant immunity through breast milk. Changes in the offspring’s immune systems, with potential effects on antimicrobial peptide and antibody production and quantity, could lead to dysregulated shaping of the microbiome in early life. We previously observed lower levels of TGF-β in the colostrum of mothers with PPD (35), which is crucial for modulating infant inflammatory responses and promoting the development of the infant’s gastrointestinal tract (36, 37). A prior study also showed that maternal depressive symptoms were linked to reduced fecal IgA concentrations in infants (10). Further studies could focus on the vertical transmission of maternal and infant microbiota in mother–child dyads with PPD. Conversely, reduced mother–infant interaction could perturb the development of infant microbiota due to reduced horizontal transmission. Maternal depression has been shown to affect the skin microbiome in offspring (38). The elucidation of the pathway by which PPD affects the offspring’s microbiome is an ongoing and crucial issue, requiring further research in both humans and animals. In addition to factors related to mother–infant bacterial transmission, the Integrative Body-Mind-Spirit (IBMS) model potentially provides an explanation for this phenomenon. From the IBMS model, which emphasize the importance of achieving balance and harmony between the body, emotions, and spirit (39, 40), a mother’s emotional status could influence the emotional and spiritual status of her infant. For example, PPD affects a mother’s emotions and life attitude, thereby diminishing her caregiving capacity and impacting the mental and neural development of her infant. Therefore, promoting the well-being of postpartum women in from Body-Mind-Spirit perspective could help mitigate the effects of PPD on their offspring.

Early-life microbial colonization plays a key role in neuropsychological development (41). Our study revealed a positive correlation between the relative abundance of *Bifidobacterium* and infant neurodevelopment. Conversely, *Finegoldia* was found to be negatively associated with infant fine motor development. These findings align with prior studies (36, 42, 43). For example, alterations in gut microbial composition have been identified in children with autism (44). Additionally, sterile mice presented more pronounced short-term cognitive and working memory impairments compared to mice with normal gut microbiota (45). These results may be linked to the brain-gut-microbiome axis, where the gut microbiome collaborates with its host to regulate the development and function of the nervous system by producing and modifying various metabolic, immunological and neurochemical factors (46). Within our co-occurrence network analysis, we observed that *Bifidobacterium* and *Finegoldia* formed co-occurring relationships with fecal L-Aspartic acid and its derivative, N-Acetyl-L-aspartic acid. *Bifidobacterium* has the potential to elevate L-Aspartic acid levels by incorporating aspartate into its peptidoglycan structure, alongside its immunomodulatory effects on brain metabolism and the synthesis of related substances within the same pathway (47–49). However, the pathophysiological mechanism linking *Finegoldia* and L-Aspartic acid remains unknown and requires further research. These findings imply synergistic relationships between altered gut microbes and the host’s metabolism in infants with maternal PPD. N-Acetyl-L-aspartic acid is a contributor to energy production from the amino acid glutamate in neuronal mitochondria, and could function as a neurotransmitter in the brain through its interaction with metabotropic glutamate receptors (50). The levels of L-Aspartic acid and N-Acetyl-L-aspartic acid have been reported to decrease in various neuropathological conditions, such as brain injury, Alzheimer’s disease, and autism (51, 52). Higher level of N-Acetyl-L-aspartic acid in the hippocampus have been associated with enhanced working memory performance in humans (53). In our study, infants whose mother had PPD exhibited a reduction in *Bifidobacterium* and L-Aspartic acid, aligning with lower neurodevelopmental scores. It is therefore important to monitor and maintain an early gut microbiota balance in infants with mothers experiencing PPD. Based on these findings, animal experiments to further identify the key gut microbial strains and their functional metabolism associated with neurodevelopment would be of paramount importance and may lead to new therapeutic strategies for mothers with PPD and their infants.

To the best of our knowledge, this study is the first to demonstrate that maternal postpartum depression symptoms may impact offspring neurodevelopment by influencing the composition of infant fecal microbiota and metabolites. This impact includes an increase in alpha diversity and a reduction in the relative abundance of beneficial bacteria, such as *Bifidobacterium*, as well as a decrease in the neurotransmitter N-Acetyl aspartic acid. Notably, our mediation analysis provided causal evidence regarding the connection between changes in the level of N-Acetyl aspartic acid and the development of infant problem-solving skills (54–56). Nevertheless, our study has several limitations. First, we did not evaluate or clinically confirm maternal depression during pregnancy and other perinatal emotional disorders such as anxiety; instead, we relied on self-reported history of depression or psychiatric diseases before or during pregnancy to exclude women with these conditions. Different mood disorders can coexist or be interrelated, and previous emotional symptoms might be associated with the occurrence of PPD. Further research could be initiated, encompassing the period from pregnancy through the postnatal phase, with a focus on gathering diagnostic data. Second, this was a single-center study encompassing approximately 100 participants, whether and to what extent the results can be generalized to other populations require confirmation by future studies in diverse populations. Third, the persistence of early PPD symptoms and how the gut microbiota evolves with age remain unclear. Therefore, longitudinal studies with larger sample sizes, multicenter designs and measuring emotional disorders before, during, and after pregnancy up to 6 months are needed to draw more definitive causal conclusions. Finally, our study did not include an analysis of maternal samples such as stool, blood, and breast milk, limiting our ability to explore potential pathways through which maternal PPD might affect maternal inflammatory and microbiome profiles, and subsequently disrupt the offspring’s microbiome. Future studies incorporating both maternal and infant microbiome samples, along with assessment of mood and behavior in both cohorts, are essential to advance our understanding of maternal influence on the infant microbiome and health.

## Conclusions

This study suggests that symptoms of maternal postpartum depression could affect infant neurodevelopment, potentially involving alterations in infant fecal aspartate metabolism. Future research focusing on mother–infant dyads over an extended period across diverse populations may reveal more direct relationships between postnatal maternal mental health and the offspring microbiome, providing further insights into how these alterations may affect offspring health outcomes.

## Data availability statement

All sequencing data associated with this study have been made publicly available. The 16S rRNA bacterial profiling data generated in this study have been deposited in the NCBI database with accession number SRA: PRJNA945351 (https://www.ncbi.nlm.nih.gov/sra). All data supporting the findings of this study are available within the paper.

## Ethics statement

The studies involving humans were approved by Ethics Committee of the Seventh Affiliated Hospital, Southern Medical University. The studies were conducted in accordance with the local legislation and institutional requirements. Written informed consent for participation in this study was provided by the participants’ legal guardians/next of kin.

## Author contributions

LZ: Conceptualization, Data curation, Formal analysis, Methodology, Writing – original draft, Writing – review & editing. LT: Formal analysis, Writing–original draft, Writing – review & editing. CZ: Writing – review & editing, Formal analysis. SW: Writing – review & editing. DK: Writing – review & editing. RX: Conceptualization, Project administration, Resources, Supervision, Writing – review & editing.
